# Safety culture in the perception of public-hospital health professionals

**DOI:** 10.11606/s1518-8787.2021055002838

**Published:** 2021-08-23

**Authors:** Paloma Aparecida Carvalho, Fábio Ferreira Amorim, Luiz Augusto Casulari, Leila Bernarda Donato Gottems

**Affiliations:** I Universidade de Brasília Programa de Pós-Graduação em Ciências da Saúde BrasíliaDF Brasil Universidade de Brasília. Programa de Pós-Graduação em Ciências da Saúde. Brasília, DF, Brasil; II Escola Superior de Ciências da Saúde Programa de Pós-Graduação em Ciências da Saúde BrasíliaDF Brasil Escola Superior de Ciências da Saúde. Programa de Pós-Graduação em Ciências da Saúde. Brasília, DF, Brasil; III Universidade de Brasília Serviço de endocrinologia Hospital Universitário de Brasília BrasíliaDF Brasil Universidade de Brasília. Serviço de endocrinologia do Hospital Universitário de Brasília. Brasília, DF, Brasil

**Keywords:** Patient Safety, Safety management, organization & administration, Patient Assistance Team, Health Knowledge, Attitudes and Practice, Evaluation of Health Programs and Projects

## Abstract

**OBJECTIVE:**

Evaluating safety culture in the perception of professionals working in public hospitals of the Unified Health System (SUS) of Distrito Federal, Brazil, three years after the implementation of the National Patient Safety Program (PNSP).

**METHODS:**

Analytical cross-sectional study conducted in eleven public hospitals using the Safety Attitudes Questionnaire (SAQ) in electronic format. Stratified sampling was estimated according to the proportion of the total number of professionals in each hospital, as well as the representativeness of each professional group. The results of the total score and domains equal to or greater than 75 were considered positive. Descriptive and inferential analyses of professional groups and hospitals were carried out.

**RESULTS:**

909 professionals participated. The total score by professional group was negative (62.5 to 69.5) and the domains differed statistically in all cases. The eleven hospitals had a negative total score (61.5 to 68.6). The domains to attain positive performance were job satisfaction, stress recognition and teamwork climate. The lowest results were in working conditions and management perception domains, for which none of the hospitals had an average above 75. Differences were also found for domain means across hospitals, except in management perception.

**DISCUSSION:**

Three years after the implementation of PNSP, the safety culture in eleven hospitals evaluated was weak, although the domains of job satisfaction, stress recognition and teamwork climate had positive results. The results can contribute to decision-making by managers, as safety culture is an essential element in the implementation of patient safety policy.

## INTRODUCTION

Unsafe care and its harmful consequences to the patient have been reported since the 1980s. The World Health Organization (WHO) launched several initiatives focused on care safety, with greater emphasis since 2004 when it created the World Alliance for Patient Safety^1^. Brazil, part of this alliance, started the construction of a patient safety policy in 2001 with the creation of the Sentinela Network, a performance and safety observatory of health products^2^.

In 2013, Brazil intensified patient safety guidelines by implementing the National Patient Safety Program (PNSP), aiming to qualify health care. The safety culture is a transversal element that permeates the four axes of the program: encouragement of safe care practice; citizen involvement in their own safety; inclusion of the theme in teaching; and increased research on the topic^1^.

The term “safety culture” is used by organizations considered to be at high risk since the Chernobyl nuclear accident^3^. In healthcare, the safety culture is described as the product of individual and group values, attitudes, perceptions, skills and behavior patterns that determine a healthcare organization’s commitment to patient safety management. Organizations with a positive safety culture are characterized by good communication between professionals, mutual trust and common perceptions about the importance of safety and the effectiveness of preventive actions^4^. The term “safety climate” is defined as the superficial and measurable characteristics of the safety culture based on the perceptions and attitudes of individuals at a given point in time^3^.

In line with international patient safety policies, PNSP follows the WHO definition of safety culture, which is based on five points: 1) all workers taking responsibility for their own safety, as well as for their colleagues’, patients’ and family members’ safety; 2) prioritizing safety over financial and operational goals; 3) encouraging and rewarding identification, notification, and resolution of safety-related issues; 4) promoting organizational learning from the occurrence of incidents; and 5) providing resources, structure and accountability for the effective maintenance of safety^1,6^.

The Unified Health System (SUS) in Brazil provides health services to the population through various establishments, from basic units to hospitals. In this system, the aim is to promote a safety culture with emphasis on organizational learning and improvement, involvement of professionals and patients in the prevention of incidents, focusing on safe systems and avoiding individual accountability processes^6^.

Knowing the perception of professionals about safety culture is an important strategy for health service managers, as it contributes to improving the quality of care and implementing PNSP. However, the use of evaluation results by decision makers remains low^7^. The aim of this study was to evaluate the safety culture according to the perception of professionals working in SUS public hospitals in Distrito Federal (DF), Brazil.

## METHODS

Analytical cross-sectional study carried out through the application of the Safety Attitudes Questionnaire (SAQ), translated and culturally adapted for Brazil^8^, which evaluates the safety climate in the perception of professionals. This study was carried out from September 2016 to January 2017. The study population consisted of professionals working in eleven public hospitals in Distrito Federal: Hospital Regional da Asa Norte, Hospital Materno Infantil de Brasília, Hospital Regional do Guará, Hospital Regional de Sobradinho, Hospital Regional de Planaltina, Hospital Regional de Brazlândia, Hospital Regional de Ceilândia, Hospital Regional de Samambaia, Hospital Regional de Taguatinga, Hospital Regional do Gama and Hospital Regional de Santa Maria. This set of hospitals totals 3,295 beds and 15,545 health professionals (Table 1). To map the installed capacity of hospitals and number of professionals, the National Register of Health Establishments (CNES) was used, according to data from May 2016. To preserve identification, each hospital was identified as HX, from H1 to H11, following the descending order of the number of beds.

**Table 1:** Number of beds and professionals in hospitals participating in the study in 2016 in Distrito Federal, Brazil.

Hospital	Beds, n	Professional, n	Sampling plan, n
H1	507	1,322	77
H2	471	1,621	88
H3	422	2,338	118
H4	409	1,847	101
H5	349	2,082	109
H6	331	1,854	101
H7	322	1,571	86
H8	166	836	52
H9	138	944	57
H10	127	681	46
H11	53	449	34
Total	3,295	15,545	869

Source: National Register of Health Establishments (CNES), May 2016.

The stratified sampling was calculated according to the proportion of the total number of professionals in each hospital and the representativeness of each professional group, with 869 professionals expected to answer the questionnaire (Table 1). The inclusion criterion was to be a professional active relationship at the hospitals participating in the study. Professionals absent during the period of data collection were excluded.

SAQ is a self-applied instrument divided into two parts. The first one consists of 41 items that comprise six domains: teamwork climate (1 to 6), safety climate (7 to 13), job satisfaction (15 to 19), stress recognition (20 to 23), management perception (24 to 29) and working conditions (30 to 32). Items 14, 33 to 36 do not belong to any domain, but make up the total score, which is calculated with all statements. The second part collects data that characterize the professionals regarding gender, profession and years of experience in the field^8^. The responses to the items follow a five-point Likert scale, with the instrument’s result varying from 0 to 100 for the total score and domains, where zero represents the worst and 100 the best perception of the safety climate. The safety climate is considered positive when the score is equal to or greater than 75 points^8^. For this research, we chose to transcribe SAQ into electronic format for mobile devices.

The team of researchers responsible for data collection attended training, awareness-raising meetings with hospital managers (directors, managers and professionals from the Patient Safety Center) and a presentation of the research project and researcher team. Subsequently, researchers visited hospitals and invited professionals to participate. Mobile devices were made available so that the Informed Consent Term (TCLE) and SAQ could be accessed.

For the analysis, professionals were divided into the following groups: 1) managers; 2) doctors; 3) nurses; 4) other graduate assistance professionals; 5) nursing technicians; 6) other assistance technical professionals; 7) non-assistance professionals from the support team.

The Kolmogorov-Smirnov test was used to assess data normality. According to their distribution, quantitative data were expressed as mean ± standard deviation (SD) or as median and interquartile range 25–75% (25–75% IQ). Categorical variables were expressed as number and percentage (%). For quantitative variables, Student’s t-test or Mann-Whitney test were used when we had two groups and the Analysis of Variance (ANOVA) or Kruskal-Wallis test for comparisons between more than two groups. For categorical variables, contingency tables and Pearson’s chi-square test (χ^2^) or Fisher’s exact test were used. *Post-hoc* analysis was performed using Student’s t-test or Mann-Whitney, with Bonferroni correction for all data with statistical significance following ANOVA or Kruskal-Wallis tests. Statistical analysis was performed using the Statistical Package for Social Sciences 20.0 Mac (SPSS 20.0 Mac, SPSS Inc., Chicago, Illinois, United States). For the results of the statistical tests, a 95% confidence level was considered.

The project was approved by the Research Ethics Committee of the Health Sciences Education and Research Foundation of the Health Department of Distrito Federal under verdict no. 1.656,350.

## RESULTS

909 professionals participated, with a mean age of 40 (SD = 10.1) years, mostly female (67.0%). Regarding professional groups, 209 physicians (23.0%), 189 nursing technicians (20.8%), 156 nurses (17.2%), 146 non-assistance professionals from the support team (16.1%) and 203 from other occupations (22.3%) participated. The mean weekly workload was 40 hours (IQ25%–75%: 40–40), and 65.1% had been working for five or more years in the hospitals where they were interviewed (Table 2). Nine professionals refused to participate.

**Table 2:** Characteristics of professionals and scores of the *Safety Attitudes Questionnaire* (SAQ) in Distrito Federal, Brazil.

Variable	
Age, years, mean (SD)	40.0 (10.1)
Sex, n (%)	
Female	609 (67.0)
Male	260 (28.6)
Did not answer	40 (4.4)
Professional group, n (%)	
Doctors	209 (23.0)
Nursing technicians	189 (20.8)
Nurses	156 (17.2)
Non-assistance professionals from the support team	146 (16.1)
Other graduate assistance professionals	119 (13.1)
Other technical-level assistance professionals	45 (5.0)
Manager	39 (4.3)
Did not answer	6 (0.7)
Hourly workload, median (IQ25%–75%)	40 (40–40)
Length of service at the hospital, n (%)	
Less than 1 year	74 (8.1)
1 to 2 years	78 (8.6)
3 to 4 years	165 (18.2)
5 to 10 years	203 (22.3)
11 to 20 years	142 (15.6)
21 years or more	124 (13.6)
Did not answer	123 (13.5)
Hospital, n (%)	
H1	77 (8.5)
H2	97 (10.7)
H3	115 (12.7)
H4	119 (13.1)
H5	111 (12.2)
H6	107 (11.1)
H7	91 (10,0)
H8	51 (5.6)
H9	63 (6.9)
H10	44 (4.8)
H11	34 (3.7)
SAQ, total score per domain, mean (SD)	64.2 (13.1)
Teamwork climate	75.0 (18.2)
Safety climate	64.4 (19.8)
Job satisfaction	79.8 (19.5)
Stress recognition	75.6 (24.8)
Management perception	55.8 (22.8)
Working conditions	50.2 (28.6)

SD: standard deviation, IQ25%–75%: interquartile range 25%–75%.

The mean total SAQ score was 64.2 (SD = 13.1; Table 2). Regarding SAQ domains, job satisfaction (79.8; SD = 19.5), stress recognition (75.6; SD = 24.8) and teamwork climate (75.0; SD = 18.2) had positive scores. Other domains had negative scores: safety climate (64.4; SD = 19.8), management perception (55.8; SD = 22.8) and working conditions (50.2; SD = 28.6; Table 3).

**Table 3:** *Safety Attitudes Questionnaire* (SAQ) and its domains by professional group in eleven public hospitals in Distrito Federal, Brazil.

	Total SAQ, mean (SD)	Teamwork climate, mean (SD)	Safety climate, mean (SD)	Job satisfaction, mean (SD)	Stress recognition, mean (SD)	Management perception, mean (SD)	Working conditions, mean (SD)
Manager	69.5 (14.5)	74.0 (20.8)	66.3 (21.6)	88.1 (13.6)	77.2 (26.3)	66.9 (24.9)	62.6 (30.1)
Medicine	64.9 (12.9)	81.4 (15.5)	64.7 (19.3)	78.4 (19.3)	78.4 (22.3)	53.2 (23.1)	46.2 (27.2)
Nursing	62.5 (13.1)	70.1 (20.5)	61.3 (19.6)	78.0 (20.9)	77.8 (23.7)	55.2 (20.1)	45.7 (27.3)
Other graduate assistance professionals	64.5 (12.9)	74.6 (17.0)	61.4 (20.7)	77.5 (21.6)	76.8 (23.8)	58.6 (21.6)	54.6 (25.7)
Nursing technicians	65.0 (12.5)	75.5 (16.9)	67.1 (18.0)	81.6 (15.2)	74.6 (25.1)	55.9 (22.6)	50.5 (28.5)
Other technical-level assistance professionals	63.1 (12.1)	77.0 (14.7)	70.0 (19.0)	77.4 (19.5)	76.5 (20.4)	48.1 (24.8)	53.0 (28.8)
Non-assistance professionals from the support team	63.0 (13.9)	70.4 (19.5)	63.3 (21.3)	80.9 (22.1)	68.0 (29.2)	57.5 (24.0)	52.5 (32.5)
p	0.067	< 0.001	0.023	0.032	0.005	0.003	0.004

SD: standard deviation.

Among professional groups, managers had the highest total SAQ scores (69.5; SD = 14.5), although groups did not differ statistically (p = 0.067). For all groups, the mean total SAQ score was below 75. Regarding SAQ domains, all professional groups differed. Job satisfaction was the only one in which all groups had an average above 75. The domains with the most unfavorable results were safety climate, management perception and working conditions, with a mean lower than 75 in all groups. In teamwork climate, physicians, nursing technicians and other technical-level assistance professionals had an average above 75. In the stress recognition, all groups had an average above 75, except for nursing technicians and non-assistance professionals from the support team (Table 3).

In teamwork climate, the *post-hoc* analysis showed that physicians had higher scores when compared to nurses [81.4 (SD = 15.5) and 70.1 (SD = 20.5); p < 0.001], to other graduate assistance professionals [81.4 (SD = 15.5) and 74.6 (SD = 17.0); p < 0.001], to nursing technicians [81.4 (SD = 15.5) and 75.5 (SD = 16.9); p < 0.001] and to non-assistance professionals from the support team [81.4 (SD = 15.5) and 70.4 (SD = 19.5); p<0.001; Table 3].

Safety climate presented a significant difference only between nursing technicians and nurses [67.1 (SD = 18.0) and 61.3 (SD = 19.6); p = 0.004; Table 3].

In job satisfaction, managers differed from physicians [88.1 (SD = 13.6) and 78.4 (SD = 19.3); p < 0.001], nurses [88.1 (SD = 13.6) and 78.0 (SD = 20.9); p < 0.001], other graduate assistance professionals [88.1 (SD = 13.6) and 77.5 (SD = 21.6); p = 0.005] and other technical-level assistance professionals [88.1 (SD = 13.6) and 77.4 (SD = 19.5); p = 0.005] (Table 3).

Stress recognition differed between physicians and non-assistance professionals of the support team [78.4 (SD = 22.3) *and* 68 (SD = 29.2); p < 0.001], among nurses and non-assistance professionals from the support team [77.8 (SD = 23.7) *and* 68.0 (SD = 29.2); p = 0.002] and among other graduate assistance professionals and non-assistance professionals from the support team [76.8 (SD = 23.8) *and* 68.0 (SD = 29.2); p = 0.007] (Table 3).

In management perception, managers differed from physicians [66.9 (SD = 24.9) and 53.2 (SD = 23.1); p = 0.001], nurses [66.9 (SD = 24.9) and 55.2 (SD = 20.1); p = 0.002], nursing technicians [66.9 (SD = 24.9) and 55.9 (SD = 22.6); p = 0.007] and other technical-level assistance professionals [66.9 (SD = 24.9) and 48.1 (SD = 24.8); p = 0.001].

Finally, in terms of working conditions, a difference was observed between managers and physicians [62.6 (SD = 30.1) and 46.2 (SD = 27.2); p = 0.001], managers and nurses [62.6 (SD = 30.1) and 45.7 (SD = 27.3); p = 0.001], other graduate assistance professionals and physicians [54.6 (SD = 25.7) and 46.2 (SD = 27.2); p = 0.007], and other graduate assistance professionals and nurses [54.6 (SD = 25.7) and 45.7 (SD = 27.3); p = 0.006; Table 3].

In all domains, SAQ scores showed statistically significant differences among the hospitals participating in the study (Table 4). Thus, the total SAQ score ranged from 61.5 (SD = 12.9) to 68.6 (SD = 10.5). Domains with the most favorable results were job satisfaction, with a mean above 75 in all hospitals, teamwork climate, which was positive in 7 hospitals, and stress recognition, with positive mean scores in 6 hospitals. The domains with the most unfavorable results were safety climate [57.7 (SD = 21.4) to 68.8 (SD = 19.1); p = 0.001], management perception [50.5 (SD = 24.4) to 59.7 (SD = 21.2); p = 0.001] and working conditions [40.9 (SD = 27.8) to 59.2 (SD = 26.7); p < 0.001], in which no hospital achieved scores higher than 75 (Table 4).

**Table 4:** *Safety Attitudes Questionnaire* (SAQ) and its domains by professional group in eleven public hospitals in Distrito Federal, Brazil.

	Total SAQ, mean (SD)	Teamwork climate, mean (SD)	Safety climate, mean (SD)	Job satisfaction, mean (SD)	Stress recognition, mean (SD)	Management perception, mean (SD)	Working conditions, mean (SD)
H1	63.5 (16.4)	75.6 (20.8)	65.6 (21.8)	76.8 (24.3)	70.5 (28.4)	57.9 (28.4)	45.3 (31.5)
H2	66.6 (16.5)	80.3 (16.5)	65.1 (19.4)	84.3 (18.1)	79.6 (24.1)	56.8 (21.5)	54.3 (31.4)
H3	61.9 (12.4)	70.6 (19.3)	58.5 (18.5)	75.8 (17.8)	75.3 (20.7)	51.8 (19.8)	45.4 (25.1)
H4	65.9 (11.7)	76.5 (17.8)	66.3 (18.2)	82.9 (17.9)	76.8 (26.3)	55.9 (21.7)	59.2 (26.7)
H5	64.2 (13.9)	76.1 (18.7)	67.5 (22.2)	79.7 (18.9)	74.0 (27.0)	53.4 (27.0)	52.6 (29.6)
H6	65.9 (13.4)	75.4 (18.5)	68.8 (19.1)	79.7 (19.9)	73.8 (24.9)	59.1 (21.6)	54.8 (28.8)
H7	61.8 (13.2)	73.8 (16.8)	64.2 (18.5)	78.0 (19.5)	77.5 (24.6)	50.5 (24.4)	46.4 (26.6)
H8	61.5 (12.9)	65.5 (18.2)	57.7 (21.4)	76.1 (23.0)	78.7 (18.7)	59.5 (21.2)	45.1 (25.0)
H9	63.2 (11.0)	75.5 (16.6)	60.1 (19.8)	80.5 (17.7)	81.3 (20.9)	58.2 (20.5)	40.9 (27.8)
H10	65.5 (13.1)	76.5 (14.4)	65.4 (19.3)	83.2 (19.3)	65.6 (30.8)	59.7 (21.2)	51.6 (31.6)
H11	68.6 (10.5)	78.2 (16.0)	66.2 (15.3)	82.1 (18.0)	75.1 (21.4)	59.2 (20.9)	45.6 (23.1)
p	0.025	< 0.001	0.001	0.036	0.041	0.073	< 0.001

SD: standard deviation.

## DISCUSSION

The perception of safety culture among professionals was negative, with a mean total SAQ score below 75. Like other studies done in Brazil^9^, the result of job satisfaction was the only one evaluated positively by all professional groups between the domains. The domains evaluated negatively by all professional groups were working conditions, management perception and safety climate, the former with the worst performance. In a study carried out in three Brazilian public hospitals, the perception of safety culture among professionals was negative, with mean scores ranging between 65 and 69, and job satisfaction was also the domain with the best evaluation^9^.

Negative evaluations of the safety culture by professionals have also been observed in other countries^13,14^. A study done in Sweden with surgical teams showed that the perception of safety attitudes was negative, except for job satisfaction, which had an average score above 75 in all professional groups – a result again similar to ours^13^. In intensive care units of ten Australian hospitals, the perception of safety culture was negative in most services, with less than half of the professionals identifying it as positive^14^. These findings suggest the need for initiatives aiming to improve the safety culture of professionals in health institutions. For example, a study in the United States found significant increases in the half-yearly follow-up of the SAQ after the implementation of programs aimed at improving quality and safety associated with a significant reduction in preventable harm, serious adverse events and adjusted hospital mortality^15^.

In the assessment by hospitals, job satisfaction also had the highest scores – a fact also observed in other Brazilian studies^9^. In other countries, job satisfaction is one of the domains with best evaluation ^13,16^. Although there was a positive evaluation in all professional groups, managers had significantly higher scores than the other groups. This aspect can be explained because, in general, managers tend to have a more positive perception of the safety culture in their institutions when compared to other professionals^20^.

The critical performance of working conditions is like that found in other studies in Brazil and other countries, being always one of the worst-evaluated domains^9^. Studies done in hospitals in Sweden^13,21^ and Australia^22^ also showed a negative perception of working conditions, but with better scores than those observed in this study. However, physicians in these countries had a more positive perception than other professionals, differing from our results.

The health system is made up of high-risk services that are still considered to be of low reliability due to the countless adverse events that continue to happen daily around the world^1,6^. In this sense, making this system more secure requires resources, structure and responsibility for the effective maintenance of safety. The participants’ perception of the precarious working conditions reflects the need for improvements in the assessed hospitals^1,23^. Furthermore, confronting working conditions results with good evaluations of job satisfaction can signal the preservation of the altruistic dimension of the health professional, which is reflected by the feeling of the social usefulness of what is produced. Interpersonal relationships, bonds of camaraderie, ways of coordination and cooperation, tacit rules of mutual help and coexistence among workers can increase job satisfaction, even in situations that are precarious for performance^24^.

Management perception was the second domain with the lowest mean among the groups of professionals and in most hospitals studied. Similar results have been reported by other studies^25,26^. In the study that evaluated the perception of nurses working with acute care in six Australian hospitals, this domain had the worst evaluation^25^. A similar situation was found in a survey carried out in Taiwan^26^. The perception of management was negative for all professional groups, and again the management group had the best score. Low scores in this domain suggest the need to improve management processes. It is essential to bring front-line professionals closer to decision makers to avoid generating a scenario in which management is not seen as a strength, but as a weakness for the safety culture, as reported in this Taiwan study^26^. Another study, also carried out in Taiwan, referred to management perception as a causal domain, as well as teamwork climate and stress recognition. Initiatives directed towards causal domains not only directly improve the domain itself, but also the performance of other domains. In management perception, other affected domains were teamwork climate, safety climate, job satisfaction and working conditions, which reinforces the importance of actions aimed at improving the management capacity of health services^27^.

Regarding patient safety, the safety climate situates the moment in which health services meet, guiding actions, promoting comparative assessment between services and monitoring results after the implementation of policies over time ^9,10,23^. Although this domain had negative mean scores in all groups, there was a more positive perception by professionals with a technical level, especially when compared to nursing technicians and nurses. In this regard, physicians have responded less positively than nurses and other assistance professionals in other studies^23,28^ – a result that deserves to be explored in further research.

Teamwork climate had a positive performance, with physicians having a significantly higher perception compared to other professional groups – which was also observed in a study carried out in two hospitals in Australia^22^. In fact, this domain has been considered a strong point by professionals involved in direct patient care^20^. As observed in another study carried out with nurses in university hospitals in Sweden^21^, in a study carried out in Slovenia, teamwork climate had the highest scores among SAQ domains^28^.

Although all domains are equally important for a safety culture, studies have shown that favorable results in teamwork climate and in safety climate are associated with lower rates of infections related to healthcare^29^. An association was also found with reduced rates of adverse event notification with teamwork climate, safety climate, working conditions and management perception. This suggests that efforts aimed at improving the perception of these domains can improve the quality of care^29^.

Stress recognition signals the professional’s ability to recognize that their performance can be influenced by stressing factors^27^. Although this domain had a positive evaluation, it was still negative in four hospitals and was worse evaluated by non-assistance professionals from the support team when compared to professionals from the front line. A previous study showed that licensed practical nurses (professionals with a secondary level of education, who circulate in the operating room) had lower mean scores than perioperative physicians and nurses. That means they were less able to recognize that their performance may be influenced by stressors when compared to other professionals^13^. An Australian study also showed that other health professionals scored lower than doctors and nurses^22^. In studies that compare different professional groups, those with less education or not directly involved in care had a more negative perception, a point that can be explored in future studies. Understanding the differences between groups of professionals is essential to direct assertive initiatives, as this domain provides a view of the professionals’ own understanding of their limitations under physical, psychological and emotional stress^8^.

One of the limitations of the study was the impossibility of randomizing participants due to the weakness of information systems about professionals in each hospital. Another limitation was not having compared the domains between hospital units, as the literature points to the existence of subcultures within the same organization ^15^. The comparison between groups of professionals in each hospital was also left out, as they go beyond the objectives of this study. Furthermore, although most studies that assessed the culture of safety have focused on the assessment of health professionals directly involved in care^13,21,23,28^, the inclusion of the manager group is important because they play a key role in promoting patient safety^30^, which also allows assessing the dissociation between the managers’ self-assessment and the health professionals’ management perception.

Safety culture assessments have taken place more frequently in recent years, and its applications are diverse, such as genuine safety climate evaluation^9,23^, assessment before and after interventions^21^ and combined measurements that seek to associate results^27,29^. Three years after the implementation of PNSP, safety culture in eleven hospitals evaluated was weak, however the domains of job satisfaction, stress recognition and teamwork climate had positive results.

We advise managers to invest in improvement initiatives, especially in areas with greater weaknesses, as they are important elements for patient safety and quality of care. The results point to fundamental issues, however, they do not cover the subject thoroughly, which requires additional studies that address the differences in the safety climate between the units that make up each hospital, as well as qualitative studies to deepen the understanding of the findings of this study.

## References

[1] 1. Ministry of Health (BR); Oswaldo Cruz Foundation; National Health Surveillance Agency. Reference Document for the National Patient Safety Program. Brasília, DF: MS; 2014 [cited 2019 Oct 19]. Available from: http://bvsms.saude.gov.br/bvs/publicacoes/documento_referencia_programa_nacional _seguranca.pdf

[2] 2. Bezerra ALQ, Silva AEBC, Branquinho NCSS, Paranaguá TTB. Analysis of technical complaints and adverse events reported in a sentinel hospital. Rev Enferm UERJ. 2009;17(18):467-72. [cited 2019 Oct 19] 17(4):467-72. Available from: https://repositorio.bc.ufg.br/bitstream/ri/15900/5/Artigo%20-%20Ana%20L%c3%bacia%20Queiroz%20Bezerra%20-%202009.pdf

[3] 3. Flin R, Burns C, Mearns K, Yule S, Robertson EM. Measuring safety climate in health care.Which Saf Health Care. 2006;15(2):109-15. 10.1136/qshc.2005.014761 PMC2464831 16585110

[4] 4. Nieva V, Sorra J. Safety culture assessment: a tool for improving patient safety in healthcare organizations. Qual Saf Health Care. 2003;12 Suppl 2:ii17-ii23. 10.1136/qhc.12.suppl_2.ii17 PMC1765782 14645891

[5] 5. Halligan M, Zecevic A. Safety culture in healthcare: a review of concepts. dimensions, measures and progress. Qual Saf Health Care. 2011;20(4):338-43. 10.1136/bmjqs.2010.040964 21303770

[6] 6. World Health Organization. Conceptual framework for the International Classification for Patient Safety: final technical report. Version 1.1. Geneva (CH): WHO; 2009 [cited 2019 Oct 19]. Available from: https://www.who.int/patientsafety/taxonomy/icps_full_report.pdf

[7] 7. Contandriopoulos AP, Rey L, Brousselle A, Champagne F. Évaluer une intervention complexe: enjeux conceptuels, méthodologiques, et opérationnels. Can J Program Eval. 2011 [cited 2019 Dec 10];26(3):1-16. Available from: https://www.ncbi.nlm.nih.gov/pmc/articles/PMC4900871/pdf/nihms2584.pdf PMC4900871 27293310

[8] 8. Carvalho REFL, Cassiani SHB. Cross-cultural adaptation of the Safety Attitudes Questionnaire - Short Form 2006 for Brazil. Rev Lat Am Enfermagem. 2012;20(3):575-82. 10.1590/S0104-11692012000300020 22991121

[9] 9. Carvalho REFL, Arruda LP, Nascimento NKP, Sampaio RL, Cavalcante MLSN, Costa ACP. Assessment of the culture of safety in public hospitals in Brazil. Rev Lat Am Enfermagem. 2017;25:e2849. 10.1590/1518-8345.1600.2849 PMC5363325 28301029

[10] 10. Oliveira ICL, Cavalcante MLSN, Aires SF, Freitas RJM, Silva BV, Marinho DMF, et al. Safety culture: perception of health professionals in a mental hospital. Rev Bras Enferm. 2018;71 Supl 5:2316-22. 10.1590/0034-7167-2018-0125 30365800

[11] 11. Luiz RB, Simões ALA, Barichello E, Barbosa MH. Factors associated with the patient safety climate at a teaching hospital. Rev Lat Am Enfermagem. 2015;23(5):880-7. 10.1590/0104-1169.0059.2627 PMC4660410 26487138

[12] 12. Rigobello MCG, Carvalho REFL, Guerreiro JM, Motta APG, Atila E, Gimenes FRE. The perception of the patient safety climate by professionals of the emergency department.Int Emerg Nurs. 2017;33:1-6. 10.1016/j.ienj.2017.03.003 28476345

[13] 13. Göras C, Unbeck M, Nilsson U, Ehrenberg A. Interprofessional team assessments of the patient safety climate in Swedish operating rooms: a cross-sectional survey. BMJ Open. 2017;7(9):e015607. 10.1136/bmjopen-2016-015607 PMC5588952 28864690

[14] 14. Chaboyer W, Chamberlain D, Hewson-Conroy K, Grealy B, Elderkin T, Brittin M, et al. CNE article: safety culture in Australian intensive care units: establishing a baseline for quality improvement. Am J Crit Care. 2013;22(2):93-102. 10.4037/ajcc2013722 23455858

[15] 15. Berry JC, Davis JT, Bartman T, Hafer CC, Lieb LM, Khan N, et al. Improved safety culture and teamwork climate are associated with decreases in patient harm and hospital mortality across a hospital system. J Patient Saf. 2020;16(2):130-6. 10.1097/PTS.0000000000000251 26741790

[16] 16. Huang CH, Wu HH, Lee YC. The perceptions of patient safety culture: a difference between physicians and nurses in Taiwan. Appl Nurs Res. 2018;40:39-44. 10.1016/j.apnr.2017.12.010 29579497

[17] 17. Smiley K, Ofori L, Spangler C, Acquaah-Arhin R, Deh D, Enos J, et al. Safety culture and perioperative quality at the Volta River Authority Hospital in Akosombo, Ghana. World J Surg. 2019;43(1):16-23. 10.1007/s00268-018-4763-y 30109388

[18] 18. Abu-El-Noor NI, Hamdan MA, Abu-El-Noor MK, Radwan AKS, Alshaer AA. Safety culture in neonatal intensive care units in the Gaza Strip, Palestine: a need for policy change. J Pediatr Nurs. 2017;33:76-82. 10.1016/j.pedn.2016.12.016 28081934

[19] 19. Buljac-Samardzic M, Wijngaarden JD, Dekker-van Doorn CM. Safety culture in long-term care: a cross-sectional analysis of the Safety Attitudes Questionnaire in nursing and residential homes in the Netherlands. BMJ Qual Saf. 2016;25(6):424-31. 10.1136/bmjqs-2014-003397 26208536

[20] 20. Odell DD, Quinn CM, Matulewicz RS, Johnson J, Engelhardt KE, Stulberg JJ, et al. Association between hospital safety culture and surgical outcomes in a statewide surgical quality improvement collaborative. J Am Coll Surg. 2019;229(2):175-83. 10.1016/j.jamcollsurg.2019.02.046 PMC6661205 30862538

[21] 21. Olsson C, Forsberg A, Bjerså K. Safety climate and readiness for implementation of evidence and person centered practice: a national study of registered nurses in general surgical care at Swedish university hospitals. BMC Nurs. 2016;15:54. 10.1186/s12912-016-0174-2 PMC5020433 27625589

[22] 22. Dunstan E, Coyer F. Safety culture in two metropolitan Australian tertiary hospital intensive care units: a cross-sectional survey. Aust Crit Care. 2020;33(1):4-11. 10.1016/j.aucc.2018.11.069 30660433

[23] 23. Yu B, Wen CF, Lo HL, Liao HH, Wang PC. Improvements in patient safety culture: a national Taiwanese survey, 2009-16. Int J Qual Health Care. 2020;32(1):A9-A17. 10.1093/intqhc/mzz099 31917449

[24] 24. Daedot P, Laval C. Común. Barcelona (ES): Gedisa; 2015. p.549

[25] 25. Soh SE, Morello R, Rifat S, Brand C, Barker A. Nurse perceptions of safety climate in Australian acute hospitals: a cross-sectional survey. Aust Health Rev. 2018;42(2):203-9. 10.1071/AH16172 28297633

[26] 26. Lee YC, Wu HH, Hsieh WL, Weng SJ, Hsieh LP, Huang CH. Applying importance-performance analysis to patient safety culture. Int J Health Care Qual Assur. 2015;28(8):826-40. 10.1108/IJHCQA-03-2015-0039 26440485

[27] 27. Lee YC, Zeng PS, Huang CH, Wu HH. Causal relationship analysis of the patient safety culture based on safety attitudes questionnaire in Taiwan. J Healthc Eng. 2018;2018;4268781 10.1155/2018/4268781 PMC5852901 29686825

[28] 28. Klemenc-Ketis Z, Deilkås ET, Hofoss D, Bondevik GT. Variations in patient safety climate and perceived quality of collaboration between professions in out-of-hours care. J Multidiscip Healthc. 2017;10:417‐23. 10.2147/JMDH.S149011 PMC5687361 29184416

[29] 29. Profit J, Sharek PJ, Cui X, Nisbet CC, Thomas EJ, Tawfik DS, et al. The correlation between neonatal intensive care unit safety culture and quality of care. J Patient Saf. 2018;16(4):e310-6 10.1097/PTS.0000000000000546 PMC6504623 30407963

[30] 30. Sammer CE, Lykens K, Singh KP, Mains DA, Lackan NA. What is patient safety culture? A review of the literature. J Nurs Scholarsh. 2010;42(2):156-65. 10.1111/j.1547-5069.2009.01330.x 20618600

