# Supplementary material for: Safety culture in the perception of public-hospital health professionals
Source: Rev Saude Publica. 2021 Aug 23;55:56. doi: 10.11606/s1518-8787.2021055002838 (PMC8386579; doi:10.11606/s1518-8787.2021055002838)
Supplement: Translation

# Translation: Cultura de segurança na percepção dos profissionais de saúde de hospitais públicos

**Authors:** Paloma Aparecida Carvalho, Fábio Ferreira Amorim, Luiz Augusto Casulari, Leila Bernarda Donato Gottems

**Affiliations:** I Brasil Universidade de Brasília. Programa de Pós-Graduação em Ciências da Saúde. Brasília, DF, Brasil; II Brasil Escola Superior de Ciências da Saúde. Programa de Pós-Graduação em Ciências da Saúde. Brasília, DF, Brasil; III Brasil Universidade de Brasília. Serviço de endocrinologia do Hospital Universitário de Brasília. Brasília, DF, Brasil

**Keywords:** Segurança do Paciente, Gestão da Segurança, organização & administração, Equipe de Assistência ao Paciente, Conhecimentos, Atitudes e Prática em Saúde, Avaliação de Programas e Projetos de Saúde

## Abstract

**OBJETIVO:**

Avaliar a cultura de segurança na percepção dos profissionais que trabalham nos hospitais públicos do Sistema Único de Saúde (SUS) do Distrito Federal, Brasil, três anos após a implantação do Programa Nacional de Segurança do Paciente (PNSP).

**MÉTODOS:**

estudo transversal analítico realizado em onze hospitais públicos por meio do Safety Attitudes Questionnaire em formato eletrônico. A amostragem estratificada foi calculada, obedecendo à proporção do total de profissionais em cada hospital, assim como a representatividade de cada grupo profissional. Os resultados do escore total e dos domínios iguais ou maiores que 75 foram considerados positivos. Realizadas análises descritivas e inferenciais dos grupos profissionais e dos hospitais.

**RESULTADOS:**

Participaram 909 profissionais. O escore total por grupo profissional foi negativo (62,5 a 69,5) e por domínio diferiram estatisticamente entre si em todos. Os onze hospitais tiveram escore total negativo (61,5 a 68,6). Os domínios com desempenho positivo foram satisfação no trabalho, percepção do estresse e clima de trabalho em equipe. Os resultados mais baixos foram condições de trabalho e percepção da gerência, e nenhum dos hospitais obteve média superior à 75 nesses domínios. Também foram encontradas diferenças nas médias dos domínios entre os hospitais, exceto em percepção da gerência.

**DISCUSSÃO:**

Após três anos de implantação no PNSP, a cultura de segurança nos onze hospitais avaliados se mostrou fragilizada, embora os domínios satisfação no trabalho, percepção do estresse e clima de trabalho em equipe tiveram resultados positivos. Os resultados podem contribuir para a tomada de decisão dos gestores, pois a cultura de segurança é um elemento essencial na implementação da política de segurança do paciente.

## INTRODUÇÃO

O cuidado inseguro e suas consequências com danos ao paciente têm sido reportadas desde a década de 1980. A Organização Mundial da Saúde (OMS) desencadeou várias iniciativas com foco na segurança do cuidado, com maior ênfase a partir de 2004 quando criou a Aliança Mundial para a Segurança do Paciente^1^. O Brasil, que integra essa aliança, iniciou a construção de uma política de segurança do paciente em 2001 com a criação da Rede Sentinela, visando atuar como observatório do desempenho e segurança de produtos voltados para a saúde^2^.

Em 2013, o Brasil intensificou suas diretrizes para a segurança do paciente ao instituir o Programa Nacional de Segurança do Paciente (PNSP) com o objetivo de qualificar o cuidado em saúde. A cultura de segurança é um elemento transversal e perpassa os quatro eixos do programa: estímulo a uma prática assistencial segura; envolvimento do cidadão na sua segurança; inclusão do tema no ensino; e incremento de pesquisa sobre o tema^1^.

O termo “cultura de segurança” vem sendo utilizado por organizações consideradas de alto risco desde o acidente nuclear de Chernobyl^3^. Na saúde, a cultura de segurança é descrita como o produto de valores, atitudes, percepções, competências e padrões de comportamento individuais e de grupos que determinam o compromisso de uma organização de saúde com a gestão da segurança do paciente. As organizações com cultura de segurança positiva são caracterizadas por boa comunicação entre os profissionais, confiança mútua e percepções comuns acerca da importância da segurança e efetividade de ações preventivas^4^. O termo “clima de segurança” é definido como as características superficiais e mensuráveis da cultura de segurança a partir das percepções e atitudes dos indivíduos em um determinado ponto do tempo^3^.

Em alinhamento com as políticas internacionais de segurança do paciente, o PNSP segue a definição de cultura de segurança da OMS, que se configura a partir de cinco características: 1) todos os trabalhadores assumem responsabilidade pela sua própria segurança, de seus colegas, dos pacientes e familiares; 2) prioriza a segurança acima de metas financeiras e operacionais; 3) encoraja e recompensa a identificação, a notificação e a resolução dos problemas relacionados à segurança; 4) promove o aprendizado organizacional a partir da ocorrência de incidentes; e 5) proporciona recursos, estrutura e responsabilização para a manutenção efetiva da segurança^1,6^.

O Sistema Único de Saúde (SUS) no Brasil disponibiliza serviços de saúde à população por meio de diversos estabelecimentos, desde unidades básicas até hospitais. Nesse sistema, busca-se promover a cultura de segurança com ênfase no aprendizado e aprimoramento organizacional, envolvimento dos profissionais e pacientes na prevenção de incidentes, foco em sistemas seguros, evitando os processos de responsabilização individual^6^.

Conhecer a percepção dos profissionais acerca da cultura de segurança é uma estratégia importante para os gestores dos serviços de saúde, pois contribui para a melhoria da qualidade dos cuidados e para implementação do PNSP. No entanto, sabe-se que o uso dos resultados das avaliações pelos tomadores de decisão ainda permanece baixo^7^. O objetivo deste estudo foi avaliar a cultura de segurança de acordo com a percepção dos profissionais que trabalham nos hospitais públicos do SUS no Distrito Federal (DF), Brasil.

## MÉTODOS

Estudo transversal analítico realizado por meio da aplicação do Safety Attitudes Questionnaire (SAQ) – traduzido e adaptado culturalmente para o Brasil^8^–, que mede o clima de segurança na percepção dos profissionais. Foi realizado no período de setembro de 2016 a janeiro de 2017. A população do estudo foi constituída por profissionais que atuam em onze hospitais públicos do DF: Hospital Regional da Asa Norte, Hospital Materno Infantil de Brasília, Hospital Regional do Guará, Hospital Regional de Sobradinho, Hospital Regional de Planaltina, Hospital Regional de Brazlândia, Hospital Regional de Ceilândia, Hospital Regional de Samambaia, Hospital Regional de Taguatinga, Hospital Regional do Gama e Hospital Regional de Santa Maria. Este conjunto de hospitais totaliza 3.295 leitos e 15.545 profissionais de saúde (Tabela 1). Para mapeamento da capacidade instalada dos hospitais e número de profissionais, foi utilizado o Cadastro Nacional dos Estabelecimentos de Saúde (CNES), conforme dados de maio de 2016. Para preservar a identificação, cada hospital foi codificado de H1 a H11, seguindo a ordem decrescente do número de leitos.

**Tabela 1:** Número de leitos e de profissionais dos hospitais participantes do estudo em 2016 no Distrito Federal, Brasil.

Hospital	Leitos, n	Profissional, n	Plano amostral, n
H1	507	1.322	77
H2	471	1.621	88
H3	422	2.338	118
H4	409	1.847	101
H5	349	2.082	109
H6	331	1.854	101
H7	322	1.571	86
H8	166	836	52
H9	138	944	57
H10	127	681	46
H11	53	449	34
Total	3.295	15.545	869

Fonte: Cadastro Nacional dos Estabelecimentos de Saúde (CNES), competência maio de 2016.

A amostragem estratificada foi calculada obedecendo à proporção do total de profissionais em cada hospital, assim como a representatividade de cada grupo profissional, sendo previstos 869 profissionais para responderem o questionário (Tabela 1). O critério de inclusão foi ser profissional com vínculo ativo com os hospitais participantes do estudo. Foram excluídos os profissionais que se encontravam afastados do trabalho no período da coleta de dados.

O SAQ é um instrumento autoaplicado dividido em duas partes A primeira é composta por 41 itens que compõem seis domínios: clima de trabalho em equipe (1 a 6), clima de segurança (7 a 13), satisfação no trabalho (15 a 19), percepção do estresse (20 a 23), percepção da gerência (24 a 29) e condições de trabalho (30 a 32). Os itens 14, 33 a 36 não pertencem a nenhum domínio, porém compõem o escore total, que é calculado com todas as afirmativas. A segunda parte coleta dados que caracterizam os profissionais quanto ao sexo, profissão e anos de atuação na área^8^. As respostas aos itens seguem escala Likert de cinco pontos, sendo que o resultado do instrumento varia de 0 a 100 para o escore total e para domínios, onde zero representa a pior e 100 a melhor percepção do clima de segurança. O clima de segurança é considerado positivo quando a pontuação é igual ou superior a 75 pontos^8^. Para esta pesquisa, optou-se por transcrever o SAQ para o formato eletrônico e disponibilizá-lo em aparelhos mobile.

Foram realizados treinamentos com a equipe de pesquisadores que realizaram a coleta, reuniões de sensibilização com gestores dos hospitais (diretores, gerentes e profissionais do Núcleo de Segurança do Paciente) e apresentação do projeto de pesquisa e da equipe de pesquisadores. Posteriormente, os pesquisadores visitaram os hospitais e convidaram os profissionais a participar. Na ocasião, foram disponibilizados aparelhos mobile para acesso ao Termo de Consentimento Livre e Esclarecido (TCLE) e o SAQ.

Para a análise, os profissionais foram divididos nos seguintes grupos profissionais: 1) gestor; 2) médicos; 3) enfermeiros; 4) outros profissionais assistenciais de nível superior; 5) técnicos de enfermagem; 6) outros profissionais assistenciais de nível técnico; 7) profissionais não assistenciais da equipe de apoio.

O Teste de Kolmogorov-Smirnov foi utilizado para avaliar a normalidade dos dados. De acordo com sua distribuição, dados quantitativos foram expressos como média ± desvio padrão (DP) ou como mediana e intervalo interquartil 25–75% (IQ25–75%). As variáveis categóricas foram expressas em número e porcentagem (%). Para variáveis quantitativas, Teste t de Student ou Mann-Whitney foi usado quando tínhamos dois grupos e o teste de Análise de Variância (ANOVA) ou Kruskal-Wallis para comparações acima de dois grupos. Para variáveis categóricas, foram usadas tabelas de contingência e teste qui-quadrado de Pearson (χ^2^) ou o teste exato de Fisher. Foi realizada análise *post hoc* com o Teste t de Student ou Mann-Whitney, com correção de Bonferroni para todos os dados com significância estatística após ANOVA ou Kruskal-Wallis. A análise estatística foi realizada por meio do Statistical Package for Social Sciences 20.0 Mac (SPSS 20.0 Mac, SPSS Inc., Chicago, Illinois, Estados Unidos). Para os resultados dos testes estatísticos foi considerado um nível de 95% de confiança.

O projeto foi aprovado pelo Comitê de Ética em Pesquisa da Fundação de Ensino e Pesquisa em Ciências da Saúde da Secretaria de Estado de Saúde do Distrito Federal sob parecer nº 1.656.350.

## RESULTADOS

Participaram 909 profissionais caracterizados pela média de idade de 40 (DP = 10,1) anos, predominantemente do sexo feminino (67,0%). Em relação aos grupos profissionais, participaram 209 médicos (23,0%), 189 técnicos de enfermagem (20,8%), 156 enfermeiros (17,2%), 146 profissionais não assistenciais da equipe de apoio (16,1%) e 203 de outras ocupações (22,3%). A carga horária semanal média foi de 40 horas (IQ25%–75%: 40–40), sendo que 65,1% trabalhavam há cinco ou mais anos nos hospitais onde foram entrevistados (Tabela 2). Nove profissionais não concordaram em participar.

**Tabela 2:** Características dos profissionais e escores do *Safety Attitudes Questionnaire* (SAQ) no Distrito Federal, Brasil.

Variável	
Idade, anos, média (DP)	40,0 (10,1)
Sexo, n (%)	
Feminino	609 (67,0)
Masculino	260 (28,6)
Não respondeu	40 (4,4)
Grupo profissional, n (%)	
Médicos	209 (23,0)
Técnicos de enfermagem	189 (20,8)
Enfermeiros	156 (17,2)
Profissionais não assistenciais da equipe de apoio	146 (16,1)
Outros profissionais assistenciais de nível superior	119 (13,1)
Outros profissionais assistenciais de nível técnico	45 (5,0)
Gestor	39 (4,3)
Não respondeu	6 (0,7)
Carga horária, mediana (IQ25%–75%)	40 (40–40)
Tempo de atuação no Hospital, n (%)	
Menos de 1 ano	74 (8,1)
1 a 2 anos	78 (8,6)
3 a 4 anos	165 (18,2)
5 a 10 anos	203 (22,3)
11 a 20 anos	142 (15,6)
21 anos ou mais	124 (13,6)
Não respondeu	123 (13,5)
Hospital, n (%)	
H1	77 (8,5)
H2	97 (10,7)
H3	115 (12,7)
H4	119 (13,1)
H5	111 (12,2)
H6	107 (11,1)
H7	91 (10,0)
H8	51 (5,6)
H9	63 (6,9)
H10	44 (4,8)
H11	34 (3,7)
SAQ, escore total por domínio, média (DP)	64,2 (13,1)
Clima de trabalho em equipe	75,0 (18,2)
Clima de segurança	64,4 (19,8)
Satisfação no trabalho	79,8 (19,5)
Percepção do estresse	75,6 (24,8)
Percepção da gerência	55,8 (22,8)
Condições de trabalho	50,2 (28,6)

DP: desvio padrão, IQ25%–75%: intervalo interquartil 25%–75%.

A média do escore total do SAQ foi 64,2 (DP = 13,1) (Tabela 2). Em relação aos domínios do SAQ, satisfação no trabalho (79,8; DP = 19,5), percepção do estresse (75,6; DP = 24,8) e clima de trabalho em equipe (75,0; DP = 18,2) apresentaram escores positivos. Os demais domínios foram negativos: clima de segurança (64,4; DP = 19,8), percepção da gerência (55,8; DP = 22,8) e condições de trabalho (50,2; DP = 28,6) (Tabela 3).

**Tabela 3:** *Safety Attitudes Questionnaire* (SAQ) e seus domínios por grupo profissional nos onze hospitais públicos do Distrito Federal, Brasil.

	SAQ total, média (DP)	Clima de trabalho em equipe, média (DP)	Clima de segurança, média (DP)	Satisfação no trabalho, média (DP)	Percepção do estresse, média (DP)	Percepção da gerência, média (DP)	Condições de trabalho, média (DP)
Gestor	69,5 (14,5)	74,0 (20,8)	66,3 (21,6)	88,1 (13,6)	77,2 (26,3)	66,9 (24,9)	62,6 (30,1)
Medicina	64,9 (12,9)	81,4 (15,5)	64,7 (19,3)	78,4 (19,3)	78,4 (22,3)	53,2 (23,1)	46,2 (27,2)
Enfermagem	62,5 (13,1)	70,1 (20,5)	61,3 (19,6)	78,0 (20,9)	77,8 (23,7)	55,2 (20,1)	45,7 (27,3)
Outros profissionais assistenciais de nível superior	64,5 (12,9)	74,6 (17,0)	61,4 (20,7)	77,5 (21,6)	76,8 (23,8)	58,6 (21,6)	54,6 (25,7)
Técnicos de enfermagem	65,0 (12,5)	75,5 (16,9)	67,1 (18,0)	81,6 (15,2)	74,6 (25,1)	55,9 (22,6)	50,5 (28,5)
Outros profissionais assistenciais de nível técnico	63,1 (12,1)	77,0 (14,7)	70,0 (19,0)	77,4 (19,5)	76,5 (20,4)	48,1 (24,8)	53,0 (28,8)
Profissionais não assistenciais da equipe de apoio	63,0 (13,9)	70,4 (19,5)	63,3 (21,3)	80,9 (22,1)	68,0 (29,2)	57,5 (24,0)	52,5 (32,5)
valor de p	0,067	< 0,001	0,023	0,032	0,005	0,003	0,004

DP: desvio padrão.

Entre os grupos profissionais, os gestores apresentaram os maiores escores totais do SAQ (69,5; DP = 14,5), não havendo, porém, diferença com significância estatística entre os grupos (p = 0,067). Vale salientar que em todos os grupos a média do escore total do SAQ foi inferior a 75. Em relação aos domínios do SAQ, houve diferença significativa entre todos os grupos profissionais. A satisfação no trabalho foi o único em que todos os grupos apresentaram média acima de 75. Os domínios com resultados mais desfavoráveis foram clima de segurança, percepção da gerência e condições de trabalho, apresentando média inferior a 75 em todos os grupos. No clima de trabalho em equipe, médicos, técnicos de enfermagem e outros profissionais assistenciais de nível técnico apresentaram média superior a 75. Na percepção do estresse, todos os grupos apresentaram média superior a 75, exceto os técnicos de enfermagem e profissionais não assistenciais da equipe de apoio (Tabela 3).

No domínio clima de trabalho em equipe, a análise *post hoc* mostrou que os médicos apresentaram valores mais elevados quando comparados aos enfermeiros [81,4 (DP = 15,5) *versus* 70,1 (DP = 20,5); p < 0,001], aos outros profissionais assistenciais de nível superior [81,4 (DP = 15,5) *versus* 74,6 (DP = 17,0); p < 0,001], aos técnicos de enfermagem [81,4 (DP = 15,5) *versus* 75,5 (DP = 16,9); p < 0,001] e aos profissionais não assistenciais da equipe de apoio [81,4 (DP = 15,5) *versus* 70,4 (DP = 19,5); p < 0,001] (Tabela 3).

O clima de segurança apresentou diferença significativa apenas entre técnicos de enfermagem e enfermeiros [67,1 (DP = 18,0) *versus* 61,3 (DP = 19,6); p = 0,004] (Tabela 3).

Na satisfação no trabalho, houve diferença significativa na comparação dos gestores com os médicos [88,1 (DP = 13,6) *versus* 78,4 (DP = 19,3); p < 0,001], com os enfermeiros [88,1 (DP = 13,6) *versus* 78,0 (DP = 20,9); p < 0,001], com os outros profissionais assistenciais de nível superior [88,1 (DP = 13,6) *versus* 77,5 (DP = 21,6); p = 0,005] e com outros profissionais assistenciais de nível técnico [88,1 (DP = 13,6) *versus* 77,4 (DP = 19,5); p = 0,005] (Tabela 3).

A percepção do estresse apresentou diferença entre os médicos e profissionais não assistenciais da equipe de apoio [78,4 (DP = 22,3) *versus* 68 (DP = 29,2); p < 0,001], entre enfermeiros e profissionais não assistenciais da equipe de apoio [77,8 (DP = 23,7) *versus* 68,0 (DP = 29,2); p = 0,002] e entre outros profissionais assistenciais de nível superior e profissionais não assistenciais da equipe de apoio [76,8 (DP = 23,8) *versus* 68,0 (DP = 29,2); p = 0,007] (Tabela 3).

Na percepção da gerência, houve diferença na comparação dos gestores com os médicos [66,9 9DP = 24,9) *versus* 53,2 (DP = 23,1); p = 0,001], com os enfermeiros [66,9 (DP = 24,9) *versus* 55,2 (DP = 20,1); p = 0,002], com os técnicos de enfermagem [66,9 (DP = 24,9) *versus* 55,9 (DP = 22,6); p = 0,007] e com outros os profissionais assistenciais de nível técnico [66,9 (DP = 24,9) *versus* 48,1 (DP = 24,8); p = 0,001].

Finalmente, nas condições de trabalho, foi observada diferença entre os gestores e os médicos [62,6 (DP = 30,1) *versus* 46,2 (DP = 27,2); p = 0,001], os gestores e os enfermeiros [62,6 (DP = 30,1) *versus* 45,7 (DP = 27,3); p = 0,001], os outros profissionais assistenciais de nível superior e os médicos [54,6 (DP = 25,7) *versus* 46,2 (DP = 27,2); p = 0,007], e os outros profissionais assistenciais de nível superior e os enfermeiros [54,6 (DP = 25,7) *versus* 45,7 (DP = 27,3); p = 0,006] (Tabela 3).

Em todos seus domínios, os escores no SAQ apresentaram diferenças estatisticamente significativas ao comparar os hospitais participantes do estudo (Tabela 4). Assim, o escore total do SAQ variou de 61,5 (DP = 12,9) a 68,6 (DP = 10,5). Os domínios com resultados mais favoráveis foram satisfação no trabalho, com média acima de 75 em todos os hospitais, clima de trabalho em equipe, que foi positivo em 7 hospitais, e percepção do estresse, positivo em 6 hospitais. Os domínios com resultados mais desfavoráveis foram clima de segurança [57,7 (DP = 21,4) a 68,8 (DP = 19,1); p = 0,001], percepção da gerência [50,5 (DP = 24,4) a 59,7 (DP = 21,2); p = 0,001] e condições de trabalho [40,9 (DP = 27,8) a 59,2 (DP = 26,7); p < 0,001], nos quais nenhum hospital alcançou resultado superior a 75 (Tabela 4).

**Tabela 4:** *Safety Attitudes Questionnaire* (SAQ) e seus domínios por hospital nos onze hospitais públicos do Distrito Federal, Brasil.

	SAQ total, média (DP)	Clima de trabalho em equipe, média (DP)	Clima de segurança, média (DP)	Satisfação no trabalho, média (DP)	Percepção do estresse, média (DP)	Percepção da gerência, média (DP)	Condições de trabalho, média (DP)
H1	63,5 (16,4)	75,6 (20,8)	65,6 (21,8)	76,8 (24,3)	70,5 (28,4)	57,9 (28,4)	45,3 (31,5)
H2	66,6 (16,5)	80,3 (16.5)	65,1 (19,4)	84,3 (18,1)	79,6 (24,1)	56,8 (21,5)	54,3 (31,4)
H3	61,9 (12,4)	70,6 (19,3)	58,5 (18,5)	75,8 (17,8)	75,3 (20,7)	51,8 (19,8)	45,4 (25,1)
H4	65,9 (11,7)	76,5 (17,8)	66,3 (18,2)	82,9 (17,9)	76,8 (26,3)	55,9 (21,7)	59,2 (26,7)
H5	64,2 (13,9)	76,1 (18,7)	67,5 (22,2)	79,7 (18,9)	74,0 (27,0)	53,4 (27,0)	52,6 (29,6)
H6	65,9 (13,4)	75,4 (18,5)	68,8 (19,1)	79,7 (19,9)	73,8 (24,9)	59,1 (21,6)	54,8 (28,8)
H7	61,8 (13,2)	73,8 (16,8)	64,2 (18,5)	78,0 (19,5)	77,5 (24,6)	50,5 (24,4)	46,4 (26,6)
H8	61,5 (12,9)	65,5 (18,2)	57,7 (21,4)	76,1 (23,0)	78,7 (18,7)	59,5 (21,2)	45,1 (25,0)
H9	63,2 (11,0)	75,5 (16,6)	60,1 (19,8)	80,5 (17,7)	81,3 (20,9)	58,2 (20,5)	40,9 (27,8)
H10	65,5 (13,1)	76,5 (14,4)	65,4 (19,3)	83,2 (19,3)	65,6 (30,8)	59,7 (21,2)	51,6 (31,6)
H11	68,6 (10,5)	78,2 (16,0)	66,2 (15,3)	82,1 (18,0)	75,1 (21,4)	59,2 (20,9)	45,6 (23,1)
valor de p	0,025	< 0,001	0,001	0,036	0,041	0,073	< 0,001

DP: desvio padrão.

## DISCUSSÃO

A percepção da cultura de segurança entre os profissionais foi negativa, com escore médio total do SAQ abaixo de 75. Semelhante a outros estudos realizados no Brasil^9^, o resultado da satisfação no trabalho foi o único avaliado de forma positiva por todos os grupos profissionais entre os domínios. Os domínios avaliados de forma negativa por todos os grupos profissionais foram condições de trabalho, percepção da gerência e clima de segurança, tendo o primeiro apresentado o pior desempenho. Em estudo realizado em três hospitais públicos brasileiros, a percepção da cultura de segurança entre os profissionais foi negativa com escores médios variando entre 65 e 69, sendo também a satisfação no trabalho o domínio que obteve a melhor avaliação^9^.

Avaliações negativas da cultura de segurança pelos profissionais também têm sido observadas em outros países^13,14^. Em estudo realizado na Suécia com equipes cirúrgicas demostrou que a percepção em relação às atitudes de segurança era negativa, exceto na satisfação no trabalho, que apresentou escore médio acima de 75 em todos os grupos profissionais – resultado novamente semelhante a esse estudo^13^. Em unidades de terapia intensiva de dez hospitais australianos, a percepção da cultura de segurança foi negativa na maioria dos serviços, sendo que menos da metade dos profissionais a identificaram como positiva^14^. Esses achados sugerem a necessidade da implementação de iniciativas que objetivem melhorar a cultura de segurança dos profissionais nas instituições de saúde. Em um estudo realizado nos Estados Unidos, por exemplo, foram observados aumentos significativos no seguimento semestral do SAQ após a implementação de programas voltados para melhoria da qualidade e segurança associados à redução significativa de danos evitáveis, de eventos adversos graves e da mortalidade hospitalar ajustada^15^.

Na avaliação por hospitais, a satisfação no trabalho também teve os maiores escores – fato também observado em outros estudos brasileiros^9^. Em estudos realizados em outros países, observa-se que a satisfação no trabalho também é bem avaliada, sendo sempre um dos domínios com melhor avaliação^13,16^. Mesmo que tenha ocorrido avaliação positiva em todos os grupos profissionais, os gestores apresentaram escores significativamente maiores que os outros grupos. Esse aspecto pode ser explicado porque, de um modo geral, os gestores tendem a apresentar uma percepção mais positiva em relação à cultura de segurança em suas instituições quando comparados a outros profissionais^20^.

O desempenho crítico das condições de trabalho é semelhante ao encontrado em outros estudos realizados no Brasil e outros países, sendo sempre um dos domínios com pior avaliação^9^. Estudos realizados em hospitais da Suécia^13,21^ e da Austrália^22^ também mostraram percepção negativa das condições de trabalho, embora com escores melhores que os observados nesse estudo. Todavia, os médicos nesses países apresentaram percepção mais positiva que os demais profissionais, contrariando os resultados aqui encontrados.

O sistema de saúde é constituído por serviços de alto risco e ainda considerados de baixa confiabilidade devido aos inúmeros eventos adversos que permanecem acontecendo diariamente em todo o mundo^1,6^. Neste sentido, torná-lo mais seguro requer recursos, estrutura e responsabilização para a manutenção efetiva da segurança. A percepção dos participantes sobre as precárias condições de trabalho reflete a necessidade de melhorias nos hospitais avaliados^1,23^. Ademais, o resultado do domínio condições de trabalho, quando confrontado com a boa avaliação da satisfação no trabalho, pode sinalizar a preservação da dimensão altruísta do profissional de saúde, que se traduz pelo sentimento da utilidade social do que se produz. As relações interpessoais, os vínculos de camaradagem, os modos de coordenação e cooperação, as regras tácitas de ajuda mútua e de convivência entre os trabalhadores podem aumentar a satisfação com o trabalho, mesmo em situações precárias para seu desempenho^24^.

A percepção da gerência foi a segunda com menor média entre os grupos de profissionais e na maioria dos hospitais examinados. Resultados similares têm sido reportados por outros estudos^25,26^. No estudo que avaliou a percepção dos enfermeiros que trabalham com cuidados agudos em seis hospitais australianos, esse domínio teve a pior avaliação^25^. Situação semelhante foi encontrada em pesquisa realizada em Taiwan^26^. Vale salientar que a percepção da gerência foi negativa por todos os grupos profissionais, sendo que novamente o grupo gestor apresentou o melhor escore. Os escores baixos nesse domínio sugerem a necessidade do aperfeiçoamento dos processos gerenciais. É fundamental aproximar os profissionais da linha de frente àqueles tomadores de decisão, a fim de minimizar a possibilidade de gerar um cenário em que a gestão não é vista como uma fortaleza, mas como uma fraqueza para a cultura de segurança, conforme relatado no estudo realizado em Taiwan^26^. Outro estudo, também realizado em Taiwan, referiu a percepção da gerência como um domínio causal, assim como o clima de trabalho em equipe e a percepção do estresse, sendo que melhorias direcionadas aos domínios causais não apenas melhoram diretamente o próprio domínio, mas também o desempenho de outros domínios. Na percepção da gerência, outros domínios afetados foram clima de trabalho em equipe, clima de segurança, satisfação no trabalho e condições de trabalho, o que reforça a importância das ações direcionadas à melhoria da capacidade gerencial dos serviços de saúde^27^.

No que diz respeito à segurança do paciente, o clima de segurança situa o momento no qual os serviços de saúde se encontram, norteando as ações, promovendo a avaliação comparativa entre os serviços e monitorando os resultados após a implementação de políticas ao longo do tempo^9,10,23^. Embora esse domínio tenha apresentado resultados negativos em todos os grupos, houve uma percepção mais positiva pelos profissionais de nível técnico, especialmente quando comparados aos técnicos de enfermagem e aos enfermeiros. Nesse aspecto, médicos têm respondido de forma menos positiva que os enfermeiros e os outros profissionais da assistência em outros estudos^23,28^ – resultado que merece ser explorado em novos estudos.

O clima de trabalho em equipe teve desempenho positivo, sendo que os médicos tiveram uma percepção significativamente maior em comparação aos demais grupos profissionais – o que também foi observado em estudo realizado em dois hospitais na Austrália^22^. De fato, esse domínio tem sido considerado um ponto forte pelos profissionais envolvidos no atendimento direto ao paciente^20^. Assim como foi observado em outra pesquisa realizada com enfermeiros em hospitais universitários da Suécia^21^, em estudo realizado na Eslovênia, o clima de trabalho em equipe apresentou os escores mais elevados entre os domínios do SAQ^28^.

Apesar de todos os domínios serem igualmente importantes para cultura de segurança, estudos têm mostrado que resultados favoráveis no clima de trabalho em equipe e no clima de segurança estão associados a menores taxas de infecções relacionadas à assistência à saúde^29^. Também foi encontrada associação com redução das taxas de notificação de eventos adversos com clima de trabalho em equipe, clima de segurança, condições de trabalho e percepção da gerência. Isso sugere que esforços voltados para melhorar a percepção desses domínios podem melhorar a qualidade do cuidado^29^.

A percepção do estresse sinaliza a capacidade do profissional reconhecer que seu desempenho pode ser influenciado por fatores estressores^27^. Embora esse domínio tenha apresentado avaliação positiva, ele ainda foi negativo em quatro hospitais e foi pior avaliado pelos profissionais não assistenciais da equipe de apoio quando comparados aos profissionais da linha de frente. Estudo anterior mostrou que enfermeiras práticas licenciadas (profissionais com formação acadêmica de nível médio, que circulam a sala de operação) tiveram pontuações médias mais baixas, que médicos e enfermeiros perioperatórios. Ou seja, elas foram menos capazes de reconhecer que seu desempenho pode ser influenciado por estressores em comparação aos outros profissionais^13^. Um estudo australiano também mostrou que outros profissionais de saúde apresentaram pontuações inferiores aos médicos e enfermeiros^22^. Nesses estudos que comparam diversos grupos profissionais, notou-se que aqueles com menor grau de formação ou não envolvidos diretamente no cuidado tiveram percepção mais negativa, fato que pode ser explorado em estudos futuros. Compreender as diferenças entre os grupos de profissionais é fundamental para direcionar melhorias assertivas, visto que esse domínio fornece uma visão do próprio entendimento dos profissionais sobre suas limitações sob estresse físico, psicológico e emocional^8^.

Uma das limitações do estudo foi a impossibilidade de randomizar os participantes pela fragilidade nos sistemas de informações sobre os profissionais em cada hospital. Outra limitação foi não ter comparado os domínios entre as unidades do hospital, visto que a literatura aponta a existência de subculturas dentro de uma mesma organização^15^. Também não foi realizada a comparação entre grupos de profissionais em cada hospital, pois extrapolam os objetivos deste artigo. Ademais, embora a maioria dos estudos que avaliaram a cultura de segurança tenha focado na avaliação dos profissionais de saúde diretamente envolvidos na assistência^13,21,23,28^, vale salientar que inclusão do grupo gestores é importante por eles desempenharem um papel fundamental na promoção da segurança do paciente^30^, o que permite ainda aferir a dissociação entre a autoavaliação dos gestores e a percepção dos profissionais sobre a gerência.

Avaliações da cultura de segurança têm acontecido com maior frequência nos últimos anos, suas aplicações são diversas como avaliação do clima de segurança genuinamente^9,23^, avaliação antes e após intervenções^21^ e aferições combinadas que buscam associar resultados^27,29^. Após três anos de implantação no PNSP, a cultura de segurança nos onze hospitais avaliados se mostrou fragilizada, no entanto os domínios de satisfação no trabalho, percepção do estresse e clima de trabalho em equipe tiveram resultados positivos.

Sugere-se que os gestores invistam em melhorias, sobretudo nos domínios com maiores fragilidades, pois são elementos importantes para segurança do paciente e qualidade do cuidado. Os resultados apontam questões fundamentais, contudo, não esgotam a discussão sobre a temática, a qual requer estudos complementares que explorem as diferenças do clima de segurança entre as unidades que compõem cada hospital, bem como estudos qualitativos para aprofundar a compreensão dos achados deste estudo.
